# The Synergistic Effects of Al^3+^ and Chitosan on the Solid–Liquid Separation of Coal Wastewater and Their Mechanism of Action

**DOI:** 10.3390/polym14193970

**Published:** 2022-09-22

**Authors:** Ming Chang, Xiaomin Ma, Xianshu Dong, Yuping Fan, Ruxia Chen

**Affiliations:** 1 Department of Mineral Processing Engineering, Taiyuan University of Technology, Taiyuan 030024, China; 2 State Key Laboratory of Mineral Processing, Beijing 100160, China

**Keywords:** coal wastewater, chitosan, solid–liquid separation, FBRM, QCM−D

## Abstract

It is important to identify an environmentally friendly and efficient flocculant that can replace polyacrylamide for the solid–liquid separation of coal wastewater. In this study, to explore whether chitosan can be used as an environmentally friendly and efficient flocculant for the solid–liquid separation of coal wastewater, AlCl_3_–chitosan was used to conduct flocculation–sedimentation and dewatering tests under different chitosan dosages and shear-strength conditions for the prepared coal wastewater. Focused beam reflectance was measured to dynamically monitor the number of refractory fine particles, and the settled flocs were photographed and analyzed with microscopy to explore the effect of AlCl_3_–chitosan on the flocculation settlement effect and floc characteristics. The synergistic mechanisms of AlCl_3_ and chitosan were investigated using quartz crystal dissipative microbalance and zeta potential measurement. The results showed that the addition of chitosan can significantly improve the flocculation–sedimentation and dewatering effects of coal wastewater. A reasonable dosage under a certain shear strength is conducive to the reduction of fine slime particles, which results in a compact floc structure, increases the floc size, and improves the settling effect. The synergistic effect of AlCl_3_–chitosan improved the electric neutralization and adsorption bridging abilities of the chitosan, and the mixed solution of AlCl_3_ and chitosan had stronger adsorption on the carbon surface. This study provides a new approach to the selection of flocculants for coal wastewater treatment.

## 1. Introduction

Water is a critical resource for life. In coal production, a large amount of water is used for mining, dust suppression, preparation, and other processes. According to incomplete statistics, 3 m^3^ of slime water is produced during mining and washing to produce 1 ton of clean coal [1]. Currently, global coal production is approximately 8 billion tons a year, which generates a large amount of coal wastewater containing large quantities of ultrafine particles [1], which are primarily composed of coal, clay minerals, and silica [2]. Coagulants and flocculants are generally added to coal wastewater for flocculation and dewatering to recover coal resources and reuse water resources [3,4]. Currently, synthetic polyacrylamide (PAM) organic flocculants and inorganic coagulants of aluminum salt and iron salt are the most widely used reagents for the solid–liquid separation of coal wastewater [5]. However, the PAM monomer acrylamide has strong neurotoxicity and carcinogenicity, and the use of PAM is associated with the risk of secondary environmental pollution [6]. Therefore, there is an urgent requirement to identify environmentally friendly and efficient alternative flocculants.

Natural polymer flocculants are favored by flocculant researchers because of their wide availability, natural nontoxicity, biodegradability, good flocculation, and green and renewable characteristics [5,7]. The majority of these studies focused on natural flocculants based on plants and marine organisms, such as guar gum [8], chitosan [9], cellulose [10], starch [11], and alginate [12]. Natural flocculants have the disadvantages of low molecular weight, low flocculation efficiency, and a narrow application range. Therefore, researchers have grafted synthetic polymers onto the skeletons of natural polymers to obtain tailored graft flocculants [5,13]. Mahto and Mishra [14] synthesized a new type of guar gum-based flocculant (GG-g-IA) for flocculation and sedimentation experiments with kaolin and coal slurry suspensions; the flocculation efficiencies of kaolin and coal powder with the optimized GG-g-IA reached 88.15% and 81.36%, respectively. Mehta [15] synthesized a new type of carboxymethyl cellulose flocculant using conventional and microwave assistance that could achieve a maximum flocculation efficiency of 83.6% on the flocculation and sedimentation of fine coal suspensions. Although the synthetic flocculant obtained by grafting can meet coal slurry treatment requirements, the cost of grafting synthetic agents is high.

The hydroxyl group is the most frequently mentioned functional group/compound that contributes to the coagulation process in biocoagulants, followed by the amine and carboxyl groups, and protein. The hydroxyl and carboxyl groups represent the ionized groups that exist in biocoagulants [16]. Certain compounds are present on specific types of biocoagulants/bioflocculants, such as chitin and chitosan. The chitosan molecular chain contains abundant amino and hydroxyl active groups, which have good flocculation, chelating, and ion-exchange properties, and are ideal cationic flocculants [17,18]. Chitosan is mainly formed through adsorption bridging, electric neutralization, and group reactions to aggregate flocculated substances into macromolecules and precipitates [17,18]. Chen [19] synthesized a new type of chelating flocculant with a branched structure by combining chitosan and PAM, and a flocculation test was performed on simulated wastewater. The strong synergistic chelation of sulfhydryl, carboxyl, amide, and hydroxyl groups was mainly used for fine particles, while the branched structure promoted the formation of large and stable flocs through adsorption and the bridging–coiling effect. Currently, research on chitosan applications focuses on printing and dyeing wastewater [20], food industry wastewater [21], heavy metal wastewater [22], domestic wastewater [23], surface water treatment [24], sludge dewatering conditioning [25,26], and other fields. Color substances, chemical oxygen demand, proteins, oils, suspended solids, and algae in the water were removed [7]. Gil [27] used chitosan to conduct flocculation and sedimentation tests on coal slurry formed by coal-fired power plants to inhibit coal dust water spraying and obtained the optimal chitosan concentration. The MTT method was used to evaluate the cytotoxicity of the flocculants, and it was determined that chitosan flocculation did not cause environmental pollution. In addition, there have been a few studies on the application of chitosan to coal wastewater.

For the flocculation and sedimentation of suspensions containing finer refractory particles, polyelectrolytes and flocculants are generally used together to improve flocculation and form larger dense flocs with regular shapes [5,28]. Zhang [29] evaluated the feasibility of integrating high-basicity polyaluminum chloride (PAC) and high-viscosity chitosan for the coagulation of low-temperature and low-turbidity water. The author speculated that larger and more settleable flocs tend to form via the synergistic effect of charge neutralization by PAC and interparticle bridging by both PAC and chitosan, leading to excellent coagulation performance. El Foulani [30] found that the combination of chitosan and polyaluminum chloride can effectively remove turbidity and natural organic matter from raw water, and the insertion of chitosan into polyaluminum chloride increased the treatment efficiency by increasing the molecular weight, contributing to bridging phenomena and charge neutralization. Wang [31] used AlCl_3_ and chitosan-induced flocculation to harvest N. ophthalmosus, and the results indicated that a higher capture yield with wider applicability could be achieved with lower dosages of the dual flocculant compared with the utilization of the two flocculants individually under most conditions.

Therefore, this study focused on coal wastewater containing a large number of difficult-to-settle fine particles, using AlCl_3_ as a coagulant and chitosan as a flocculant compound for the flocculation sedimentation dehydration test to explore whether chitosan can meet the requirements for solid–liquid separation of coal slurry. Flocculation, sedimentation, and dewatering experiments of coal slime wastewater with different chitosan dosages and different shear strength conditions were performed. The number of fine particles in the upper clarification layer of coal wastewater during sedimentation was dynamically monitored by focused beam reflectance measurement (FBRM), and the floc morphology after sedimentation was photographed and analyzed using microscopy to explore the influence of AlCl_3_ and chitosan on the flocculation–sedimentation effect and floc characteristics of coal wastewater. The adsorption behavior of chitosan on the surface of the carbon sensor was tested using a quartz crystal dissipative microbalance (QCM−D) combined with the potential change test of coal slime particles, and the synergistic mechanism of AlCl_3_ and chitosan was analyzed. This study provides a new approach for selecting flocculants for coal wastewater treatment.

## 2. Materials and Methods

### 2.1. Materials and Reagents 

#### 2.1.1. Coal

**Figure 1:**
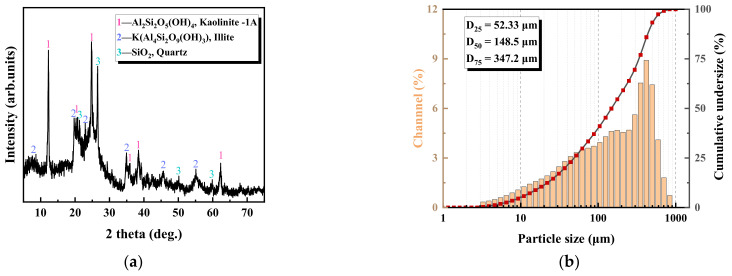
X-ray diffraction pattern (**a**) and laser particle size distribution (**b**) of lignite coal.

Fine-grained coal samples from a lignite sorting plant in Bayingol, Inner Mongolia, China, were used and dried for sample characterization. Table 1 shows the industrial and elemental analyses of coal. Figure 1a shows the XRD results of the coal samples; the minerals contained in the coal samples include kaolinite, illite, and quartz. Among them, kaolinite tends to be cloudy in water, which causes difficulties in settling coal slurry [32]. Figure 1b shows the cumulative particle size distribution curves of the coal samples, indicating that the D_25_, D_50,_ and D_90_ of the coal particles were 52.33, 148.5, and 347.2 μm, respectively. 

**Figure 2:**
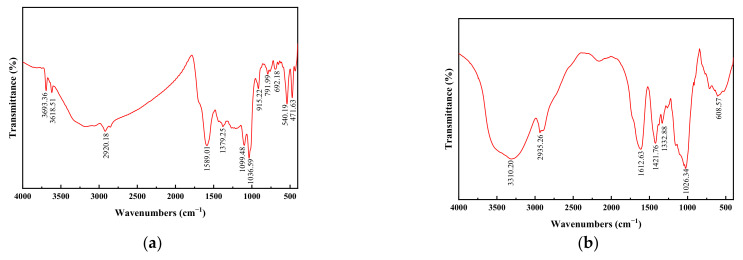
Infrared spectrogram of the coal sample (**a**) and chitosan (**b**)**.**

It can be seen from the FTIR spectra of the coal samples in Figure 2a that numerous absorption spikes appear at 3693.36 and 3618.51 cm^−1^, which correspond to the absorption band of the telescopic vibration of hydroxy-OH, representing the telescopic vibration of hydroxy-OH in coal sample absorption; the peaks at 3100–2800 cm^−1^ represent the telescopic vibrations of aromatic C–H and aliphatic C–H in the sample. Two absorption peaks appeared at 1589.01 and 915.22 cm^−1^, representing the stretching vibration of aromatic carbonyl (C=C), bridge (C=O), and carboxylate. The peak at 1036.59 cm^−1^ represents the C-O of phenols, alcohols, and ethers. The peak at 540.19 cm^−1^ represents aromatic disulfide -SS-. This indicates that the lignite sample is rich in oxygen-containing functional groups.

#### 2.1.2. Chitosan

1 g chitosan (deacetylation degree ≥90%, viscosity 100–200 mPa·s; Sinopharm Group Chemical Reagent Co., Ltd., Shanghai, China) solid powder was dissolved in 100 mL of 1% glacial acetic acid solution with a magnetic stirrer for 24 h. The volume was fixed at 1000 mL as a stock solution with a concentration of 1 g/L chitosan. Aluminum chloride (AR grade) was purchased from Aladdin (Shanghai, China). Milli-Q water was used in this study.

Figure 2b shows the FTIR spectrum of the chitosan used in the experiment. The broad set of IR absorption peaks at 3410.20 cm^−1^ corresponded to the O-H and NH_2_ stretching vibration peaks. The two strong absorption peaks at 2935.26 and 1612.8 cm^−1^ corresponded to the asymmetric stretching vibrations of methyl-CH_2_ in monosubstituted amides and the N–H bending vibration of amino groups in chitosan, respectively. The absorption peaks at 1421.1, 1332.88, 1208.2, and 1026.34 cm^−1^ corresponded to the C=O stretching vibration of a carboxyl group, the C–N stretching vibration of an amide group, the C–O stretching vibration, and the C–N stretching vibration of an amino group in the polymer, respectively. Meanwhile, there was a large absorption spike at 608.57 cm^−1^ for the absorption peak generated by the -O- and -OH- bending vibrations [26]. This is consistent with the structural formula of chitosan. 

### 2.2. Methods 

#### 2.2.1. Flocculation and Sedimentation Experiments

The coal sample (2.5 g) was stirred with approximately 250 mL of ultrapure water to prepare a coal suspension with a concentration of 10 g/L at its nature pH (7.4), which was transferred to a 250 mL settling tube. Coagulant AlCl_3_ (1 mL) at a concentration of 100 mM was added, and the settling tube was rotated for 30 s to completely mix the coagulant and suspension. After 30 s, the flocculant chitosan solution was added, and the settling tube was rotated for 30 s to completely mix the chitosan. Next, the settling tube was placed upright on the test bench, and the time was immediately maintained to record the position of the falling clarified liquid surface. In this experiment, the settlement velocity was calculated by observing the drop position at the clarified liquid level. After 5 min, the thickness of the compression layer was recorded, and the supernatant was collected. The transmittance and absorbance were measured using a UV spectrophotometer. 

#### 2.2.2. Filtration Experiments 

The filtration experiments were performed using the Büchner funnel filtration apparatus according to Fan et al. [33]. All prepared coal slurry water samples had a solid concentration of 40 g/L. Before the filtration experiment, flocculation and sedimentation were performed on the coal slime water samples according to the conditions of the previous flocculation sedimentation experiment. The procedure is summarized as follows: First, the vacuum pump was turned on, and the pressure was set to 0.08 MPa, and then at the end of the sedimentation test, a coal slurry sample was rapidly poured into the Büchner funnel. Standard quantitative filter paper with a 2 µm pore size and 9 cm diameter was used. The filtrate volume and time interval were recorded, and the filtration and dewatering rates were calculated according to Equations (1) and (2). The test was terminated by pumping until the upper layer of the filter cake was water-free and maintained for 30 s. The moisture content of the filter cake was determined. As described in Equation (3), Ruth’s theory [34] was used to determine the average mass specific resistance of the filter cake:

(1) $$v_{i}=\frac{V_{i}-V_{i-1}}{\left(t_{i}-t_{i-1}\right)\cdot \pi \cdot r^{2}}$$

where $v_{i}$ is the filtration rate, mL/s·cm^2^; $V_{i}$ is the volume of filtrate to be filtered, mL; $t_{i}$ is the dewatering time at filtrate volume $V_{i}$, s; and r is the radius of the Büchner funnel, cm.

(2) $$u=\frac{V}{t\cdot \pi \cdot r^{2}}$$

where u is the dewatering rate, cm/min; V is the volume of the filtrate when the visible water disappears from the filter cake surface, mL; t is the dewatering time when the visible water disappears from the filter cake surface, min; and r is the radius of the Büchner funnel, cm.

(3) $$v=\frac{dV}{Adt}=\frac{∆P\left(1-ms\right)A}{\mu \alpha_{av}\left(V+V_{m}\right)\rho s}$$

where v is the filtration rate, m/s; V is the filtrate volume, m^3^; $V_{m}$ is the equivalent filtrate volume, m^3^; t is filtration time, s; A is the effective filtration area, m^2^; μ is the viscosity of the filtrate, mPa·s; ∆P is the filtration pressure, Pa; m is the solid/water ratio, %; s is the solid concentration, kg/L; ρ is the density of the filtrate, kg/m^3^; and $\alpha_{av}$ is the average specific cake resistance, m/kg. The calculation procedure has been previously described [35]. 

#### 2.2.3. FBRM Test

The in situ measurement of the chord length of aggregates was performed using a Mettler-Toledo G400 FBRM probe to monitor the real-time evolution of particle agglomeration after reagent addition [36]. Chord length data can reflect changes in particle/floc size and particle/floc number in real time. The not weighted and squared weighted chord lengths measured by the FBRM probe and applied to the chord length distribution reflect the direct and volumetric chord lengths of the particles, respectively. The chitosan-formed coal slurry flocs were relatively large and settled easily; therefore, FBRM was used to monitor the variations in hard-to-settle fine coal particles in the upper clarification layer of coal wastewater. The test range was 1–500 μm. 

In this experiment, the FBRM probe was inserted into a 300 mL glass beaker containing the same 250 mL of coal slurry suspension as that in the sedimentation test, and the probe was placed 2 cm from the surface of the coal slurry. The test time was set to 30 min, and the value was recorded every 2 s. The stirring speed was 150 rpm, and 1 mL of 100 mM AlCl_3_ solution was added after stirring for 5 min. Different doses of chitosan were added to the suspension after 10 min, stirring was stopped after 15 min, and the suspension was allowed to settle for 5 min. For different shear strengths, the stirring speed and stirring time were adjusted as required.

#### 2.2.4. Optical Microscope Observations 

**Figure 3:**
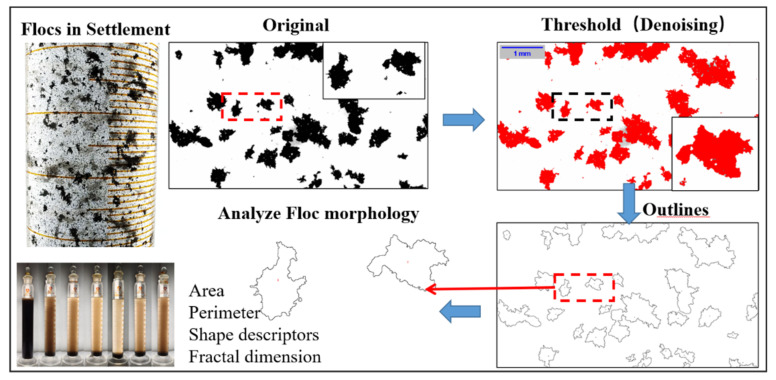
Flow of the morphological analysis of coal slurry flocs.

The sizes and structures of the coal slurry particles and flocs were observed using a Nikon SMZ745T body microscope (Tokyo, Japan). According to the procedure described above, the flocs in the compression layer were gently removed from the glass slide with a 5 mm diameter glue tip dropper and then diluted for even dispersion. The glass slide was left to dry to prevent liquids from causing reflections during shooting. The flocs were observed and photographed under a 20× objective lens under different conditions, and the number of flocs photographed under each condition was ≥80. The microscopic images of flocs without overlap were analyzed using ImageJ software (Version 1.53e, Wayne Rasband and contributors, National Institutes of Health, Bethesda, MD, USA) through black-and-white binarization. The floc profile area, equivalent diameter, morphological properties, and fractal dimensions were measured. Fractal theory is the most suitable theory for describing the morphology of agglomerates because of the nonlinear, random, and dissipative nature of the particle–agglomerate system during its evolution. Fractal dimension and lacunarity, an ImageJ plug-in for analyzing fractals, was used to calculate the fractal dimensions of flocs based on the box-counting method. Figure 3 shows the flow of the coal slurry floc morphology analysis. The effective density of flocs was calculated by taking a 500 mL measuring cylinder with a rubber-tipped burette and recording the settling process of single flocs according to the Stokes formula (Equation (4)) [37]:

(4) $$∆\rho =\rho_{f}-\rho_{w}=\frac{18\mu v_{f}}{gd^{2}}$$

where ∆ρ is the effective density, kg/m^3^; $\rho_{f}$ and $\rho_{w}$ are the densities of the floc and water, respectively, kg/m^3^; μ is the dynamic viscosity coefficient of water, Pa·s; g is the gravitational acceleration, m; $v_{f}$ is the settling velocity, m/s; and d is the floc size, m. In this study, the Reynolds coefficient was ignored for the static settling behavior of the flocs.

#### 2.2.5. QCM−D Text

In this study, the adsorption and desorption states of AlCl_3_ and chitosan on an amorphous carbon sensor surface were investigated using a QCM−D system. QCM−D measurements were performed on a QSense^®^ analyzer (Biolin Scientific, Stockholm, Sweden) using a carbon sensor (300 nm thickness, QSX 999) purchased from NanoScience Instruments (Phoenix, AZ, USA). After assembling the carbon sensor during typical measurements, the solution was tested using an Ismatec digital peristaltic pump (Opfikon, Switzerland) to continuously flow the solution across the sensor surface at a 0.1 mL/min rate at 25 °C. Before starting the measurements, the manufacturer’s sensor cleaning protocol was followed, which was essential for obtaining a stable baseline and reproducibility between measurements [38]. The water adsorption curve is accepted as a baseline (zero point) by first passing in ultrapure water and waiting for the frequency (f) and dissipation (D) to stabilize (typically, the third frequency is −382 Hz and the fifth is −179 Hz), and then starting to pass the 100 mM AlCl_3_ solution for measuring. When the solution touches the sensor surface, f and D will change; the change will typically take 20 min to stabilize. Next, chitosan should be added at a concentration of 100 mg/L dissolved in 100 mM AlCl_3_ background solution after the adsorption is stable. Water should then be injected to clean the surface, and the test must be stopped when f and D are stable again. The collected frequency and dissipation data were analyzed using the commercial software package Dfind (Version 1.2.7, Biolin Scientific, Gothenburg, Sweden) and the standard Smartfit model to calculate the adsorption or desorption mass per unit area based on the frequency change. The third overtone frequency was reported for the frequency shift and dissipation data plots.

#### 2.2.6. Zeta Potential Measurements

The main purpose of the zeta potential measurements was to study the effects of Al^3+^ and chitosan on the surface electrical properties of the coal slurry particles. The zeta potential of the coal slurry particles in the mixed stable suspensions before and after dosing was measured using a Zetasizer Ultra (Malvern Panalytical, Malvern, UK) potential analyzer. Each sample was tested three times in parallel, and the average was taken as the sample zeta potential.

## 3. Results and Discussion

### 3.1. Solid–Liquid Separation Test Results

#### 3.1.1. The Need for AlCl_3_

**Figure 4:**
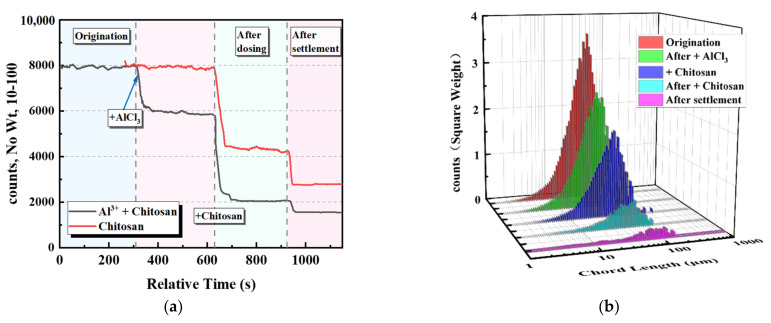
Effects of AlCl_3_ on the dynamic process of microfine particles in the upper clarification layer. (**a**) Dynamic change process of 10–100 μm particles in the supernatant. (**b**) Chord length distribution of the supernatant particles in the range of 1–500 μm.

After the laboratory test, the settling velocity of the coal slurry was accelerated by adding chitosan to the coal slurry water alone. However, there were multiple residual microfine particles, predominantly 10–100 μm particles. The upper clarification layer was turbid; therefore, it was necessary to conduct compound experiments with coagulant AlCl_3_ to study the coagulation–flocculation effect of chitosan on coal slurry water [17]. The results are shown in Figure 4. After adding AlCl_3_, the coal slurry particles were agglomerated, and the number of 10–100 μm particles was reduced by 25%. When chitosan was added to the coal slurry water for flocculation and sedimentation, the settlement effect was considerably better than that of chitosan alone. The number of particles in the upper layer after settling was 40% of that without the addition of AlCl_3_. To study the effect of chitosan, subsequent tests were conducted by adding 1 mL of AlCl_3_ solution at a concentration of 100 mM.

#### 3.1.2. Result of the Flocculation and Sedimentation Experiments

**Figure 5:**
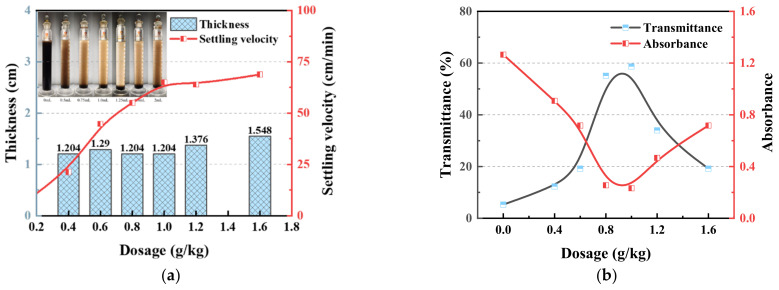
Effects of different dosages of chitosan on the sedimentation effect and clarity of coal slurry water. (**a**) The settling velocity and thickness of the compressed layer. (**b**) Transmittance and absorbance.

Figure 5 shows the effects of different dosages of chitosan on the sedimentation of the coal slurry water. The evaluation indices include the settling velocity, thickness of the compressed layer (Figure 5a), and clarity of the upper clarification layer (Figure 5b). It can be seen that the addition of chitosan significantly improved the settling velocity of the coal slurry water. The settling velocity increased rapidly with an increase in chitosan dose. When the drug dose was increased to 1.0 g/kg, the settling velocity reached 65 cm/min, and then the increasing trend slowed. This was significantly better than the experimental results obtained by Sabah and Erkan [39] using anion, cation, and non-polyacrylamide to conduct flocculation sedimentation tests on Tunçbilek coal preparation plant wastewater and better than Hansdah’s [40] and Kumar’s [41] settling effects of coal fine tailings using anionic polyacrylamide. The thickness of the compressed layer is an important index for evaluating the conversion of coal sludge water to dewatered treatable materials. With an increase in the chitosan dosage, the thickness of the compressed layer first slightly decreased, and then somewhat increased, and was maintained at approximately 1.2 cm, which is related to the floc characteristics formed by the action of chitosan. The clarity of the supernatant first improved rapidly with the increase in drug dose and then decreased, reaching the best clarity at a chitosan dose of 1.0 g/kg, with 58.7% transmittance and 0.232 absorbance. This is consistent with the experimental phenomena obtained by Sabah and Erkan [39]. Because of the increased chitosan dosage, the formation speed of the large flocs increased and settled in advance, the flocculation time of the fine particles in contact with chitosan was shortened, and a large number of fine particles remained in the upper layer. This caused the macroscopic settling velocity to increase, and the clarity was low. The best sedimentation effect was achieved only at an appropriate chitosan dosage. Compared with the flocculation effects of polytitanium tetrachloride, titanium tetrachloride (TiCl_4_), and commonly used ferric chloride (FeCl_3_) coagulants [42], the chitosan used in this study has the advantages of high flocculation rate and only slight consumption of chemicals.

#### 3.1.3. Results of the Filtration Experiments

**Figure 6:**
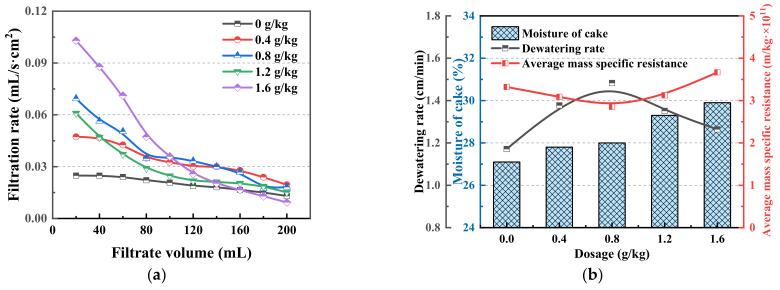
Effect of different chitosan dosages on the dewatering effect of coal slurry water. (**a**) The filtration rate. (**b**) Dewatering rate, average mass specific resistance, and filter cake moisture.

Figure 6 shows the effects of different dosages of chitosan on the dewatering effect of the coal slurry water. The evaluation indicators included filtration rate, dewatering rate, average mass specific resistance, and filter cake moisture. Filtration rate is an important index for evaluating the dewatering process of slime flocs. It can be seen from Figure 6a that with an increase in filtrate volume, the filtration rate of coal slime decreases gradually under different chitosan dosages. With an increase in dosage, the initial filtration rate of coal slime improved, and the decrease in filtration rate in the later period increased. Filtration is the process of coal slurry filter cake formation. Under the action of chitosan, the coal slime particles flocculate into clusters and settle at the bottom to form a skeleton with a porous structure to facilitate dewatering. In the later filtration stage, the fine particles migrate into the filter cake pores, and filter cake dewatering becomes difficult under pressure compression deformation [43]. An increase in the dosage affects the structure of the floc and the viscosity of the slime water, thereby affecting the entire filtration process and the properties of the filter cake [26]. With an increase in the dosage of chitosan, the moisture content of the filter cake increased, the dewatering rate first increased and then decreased, and the average mass specific resistance first decreased and then increased. The dewatering effect was the best when the dosage was 0.8 g/kg, the moisture content of the filter cake was 28%, the dewatering rate was 1.49 cm/min, and the average mass specific resistance was 2.853 × 10^11^ m/kg. A filter cake with $\alpha_{av}$ value in the range of 10^11^–10^13^ belongs to the medium filtration resistance filter cake, similar to the dewatering effect obtained by Fan [33] using polyacrylamide as a flocculant for flocculation sedimentation and filtration dewatering of fine coal. The results of this test are also consistent with Shi’s [44] conclusion that chitosan has excellent performance in improving sludge dewatering based on using chitosan to dewater sludge.

### 3.2. Effect of Different Chitosan Dosages on the Fine Particles and Floc Characteristics

#### 3.2.1. Dynamic Change Process of Fine Particles

**Figure 7:**
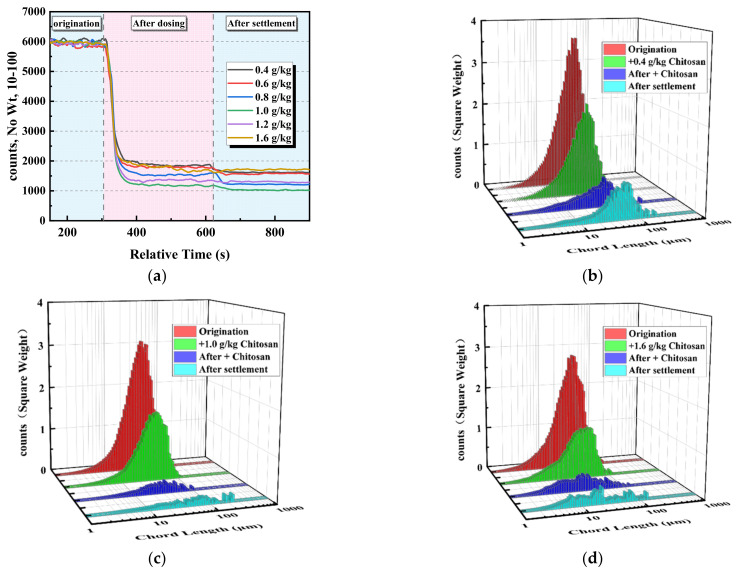
Dynamic monitoring of the effect of chitosan dosage on fine particles. (**a**) Dynamic change process of the number of 10–100 μm particles in the supernatant under different chitosan doses. (**b**–**d**) Chord length distribution of the supernatant particles in the range of 1–500 μm at chitosan doses of 0.4, 1.0, and 1.6 g/kg.

Different chitosan doses can promote the sedimentation of coal slime water, and the clarity of the supernatant is greatly affected by changes in chitosan dose. Therefore, using FBRM at a stirring speed of 150 rpm to study the change in the amount of difficult-to-sink fine particles in the upper part of the beaker with the chitosan, we explored how to adjust the chitosan dose to further reduce the residual particles and improve the clarity of the supernatant. Figure 7a shows the dynamic change process of the number of 10–100 μm particles in the supernatant under different chitosan doses. Figure 7b–d show the chord length distributions of the supernatant particles in the range of 1–500 μm under different chitosan doses at each stage of dosing, where red represents the square weight before the chitosan dosing, green during the chitosan drug action process, blue after the chitosan dosing stabilization, and cyan is after the stirring has been stopped and the settlement has stabilized.

After adding the chitosan agent, the fine particles of coal slurry rapidly adhered to each other, flocculated into larger flocs, and settled. The number of fine particles decreased rapidly, the peak of the chord length distribution during the drug action process decreased, and the peak position shifted slightly upward. The peak of the chord length distribution after the stabilization of chitosan dosing decreased, the peak position shifted downward, and the upper part of the beaker contained more fine particles that had not settled. After the stirring stopped and the settlement stabilized, the peak of the chord length distribution increased, and the peak position shifted slightly upward, which was caused by the uplifting of the fine particles in the middle of the beaker at the end of stirring.

After the stabilization of chitosan, the number of remaining particles in the upper layer of the beaker first decreased and then increased with increasing drug dose. The number of remaining particles was lowest when the drug dose was 1.0 g/kg, which indicated that these were the best conditions for floc formation. The low drug dose was insufficient for adequate particle contact, while a high dosage of flocculation and sedimentation was too fast, and the efficacy of the drug was not fully exerted. After stopping stirring and settling, the number of particles continued to decrease at a lower drug dose of 1.0 g/kg. In comparison, the number of particles was mostly unchanged under the drug doses of 1.2 and 1.6 g/kg, which was caused by the formation of more tiny flocs with slow settling under the low drug dose. The test results are consistent with the sedimentation test results.

#### 3.2.2. Floc Morphology and Properties

**Figure 8:**
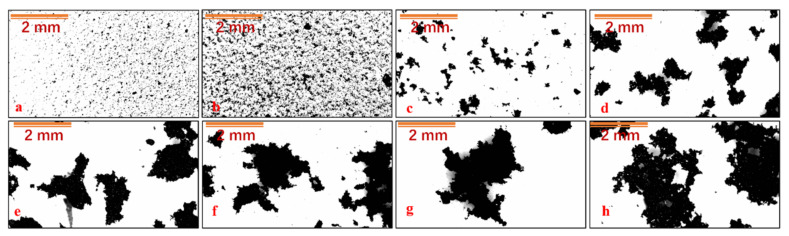
Morphology of coal slurry flocs under different chemical conditions. (**a**) No drug, (**b**) Al^3+^, (**c**) Al^3+^ + 0.4 g/kg chitosan, (**d**) Al^3+^ + 0.6 g/kg chitosan, (**e**) Al^3+^ + 0.8 g/kg chitosan, (**f**) Al^3+^ + 1.0 g/kg chitosan, (**g**) Al^3+^ + 1.2 g/kg chitosan, and (**h**) Al^3+^ + 1.6 g/kg chitosan.

**Figure 9:**
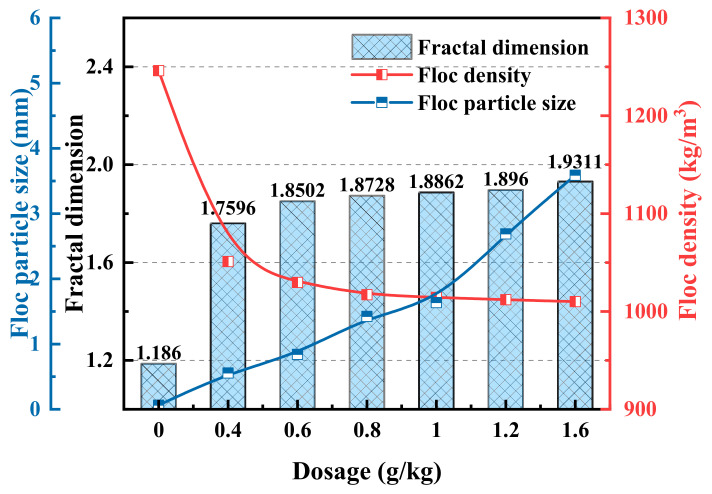
Characteristics of coal slurry flocs under different chemical conditions.

Figure 8 shows the morphology of the coal slurry flocs under different chemical conditions, and Figure 9 shows the characteristics of the coal slurry flocs under different chemical conditions after image processing and analysis. The agglomeration of coal slurry particles occurs under the action of Al^3+^, and with the addition of chitosan, flocs are formed. Chitosan has an obvious effect on the flocculation of coal slurry particles and a floc size of >1 mm. Comparing the floc characteristics of 0.8 and 1.6 g/kg chitosan, the floc size was 1.423 mm at 0.8 g/kg and 3.588 mm at 1.6 g/kg, the fractal dimension of floc increased from 1.87 to 1.93, and the floc density decreased from 1017 kg/m^3^ to 1010 kg/m^3^.

**Figure 10:**
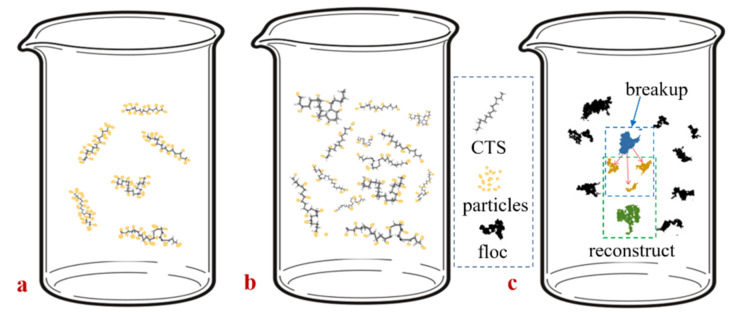
Comparison of coal slurry flocculation at high and low chitosan doses: (**a**) low-dose, (**b**) high-dose, (**c**) small flocs flocculate and form larger flocs.

Figure 10 shows the flocculation formation at high and low chitosan doses. At low dosages, the adsorbable particle sites of chitosan adhere to the particles and flocculate to form small, relatively dense flocs, so that chitosan has no remaining adsorption sites to flocculate with other small flocs. As the dosage increased, the amount of chitosan in the beaker increased, and the more spatial locations where flocculation of coal sludge particles occurred, more small flocs formed at the beginning of the increase in the beaker; additionally, the network structure of the formed flocs had a high degree of spatial dispersion and high porosity. Simultaneously, there were more sites of the remaining unabsorbed chitosan particles, and the flocs collided and adhered to the small flocs with a high success rate during the water flow and settling processes. The formed flocs had more edge branches and were irregular, larger in size, and increased in fractal dimension. Although floc porosity increased, the overall settling speed of pore-wrapped water increased, and the calculated reduction in floc density was not significant [45].

### 3.3. Effect of Different Shear Strengths on the Microfine Particles and Floc Characteristics

#### 3.3.1. Dynamic Change Process of Microfine Particles

The above test results show that chitosan meets the requirements of an agent for the solid–liquid separation of coal slurry water. Stirring and shearing can improve the collision between the agent and the particles to promote flocculation and also cause the floc to break [46]. To improve the settling effect of chitosan on difficult-to-settle fine particles and explore the effect of chitosan on floc strength, FBRM was used to study the dynamic change process of the number of fine particles in the upper part of the beaker under different shear strengths. The stirring speed was first stabilized at 150 rpm and then altered by adding 1.2 g/kg chitosan at 200, 300, 400, 500, and 600 rpm. After maintaining the speed for 5 min, stirring was stopped and the slime settlement was allowed to occur.

**Figure 11:**
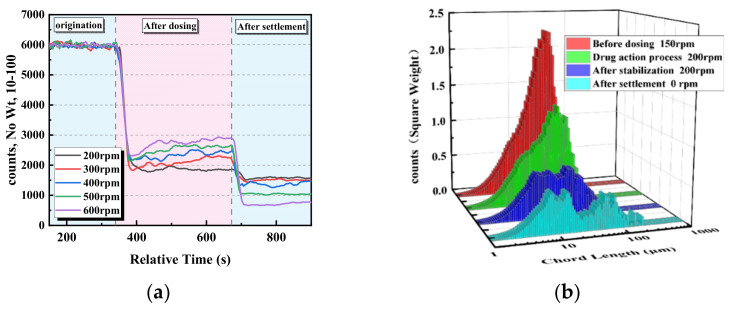

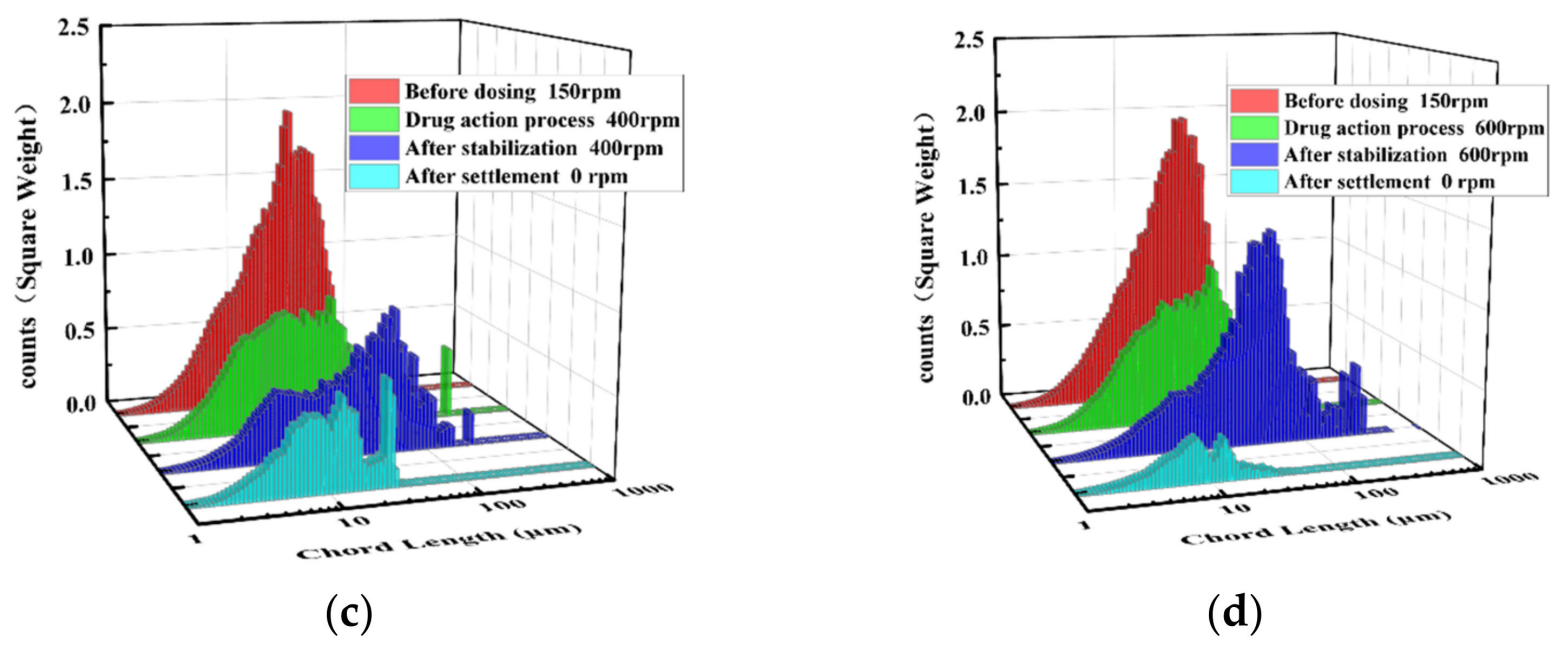
Dynamic monitoring of the effects of different stirring speeds on fine particles. (**a**) Dynamic changes in the number of 10–100 μm particles in the supernatant at different stirring speeds. (**b**–**d**) Chord length distribution of the supernatant particles in the range of 1–500 μm at 200, 400, and 600 rpm.

Figure 11a shows the dynamic change process of the number of 10–100 μm particles in the supernatant under different stirring speeds. After adding the chitosan, the fine particles of coal slurry rapidly adhered to each other and collided to flocculate into larger flocs and settle, and the number of particles rapidly decreased. After the stabilization of chitosan dosing, the number of remaining particles in the upper layer of the beaker increased with increasing stirring speed, and the higher the stirring speed, the greater the number of microfine particles. It should be the increase in shear strength that causes coal slurry flocs to break and fine particles to increase. After the stirring was stopped and the settling occurred, a higher stirring speed resulted in fewer microfine particles. The flocculation process of fine coal slurry particles can be divided into aggregation, fragmentation, and substabilization stages.

Figure 11b–d show the chord length distributions of the supernatant particles in the range of 1–500 μm under different stirring speeds in each stage of dosing, where red is for before the chitosan dosing, green is during the chitosan drug action process, blue is after the chitosan dosing stabilization, and cyan is after the stirring was stopped and settling took place. During the drug action process, the peak value of the chord length distribution decreased and the peak position moved upward. With an increase in the stirring speed, the decreasing amplitude of the peak value of the chord length distribution during the dosing reaction first increased and then decreased. After chitosan dosing stabilization, the peak of the chord length distribution at low shear intensity decreased and the peak position shifted upward, while the peak value of the chord length distribution at high shear intensity increased and the peak position shifted more strongly. This indicated that an appropriate increase in shear could strengthen the collision of fine particles and form larger flocs to promote flocculation and sedimentation. With an increase in the shear intensity, the flocs were broken up and dissociated into larger floc fragments in the upper part of the beaker to affect flocculation. After the stirring was stopped and settling took place, with the increase in stirring speed, the peak value of the chord length distribution decreased, and the peak position moved downward, which indicates that although the enhancement of shear strength caused an increase in fragmentation, it was ultimately beneficial to the reduction of microfine particles.

#### 3.3.2. Floc Morphology and Properties

**Figure 12:**
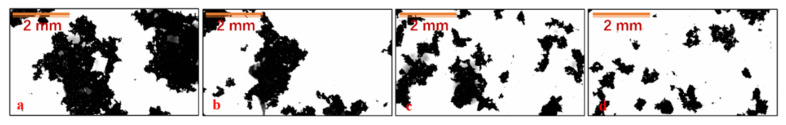
Morphologies of coal slurry flocs at different stirring speeds: (**a**) 150 rpm, (**b**) 200 rpm, (**c**) 400 rpm, and (**d**) 600 rpm.

**Figure 13:**
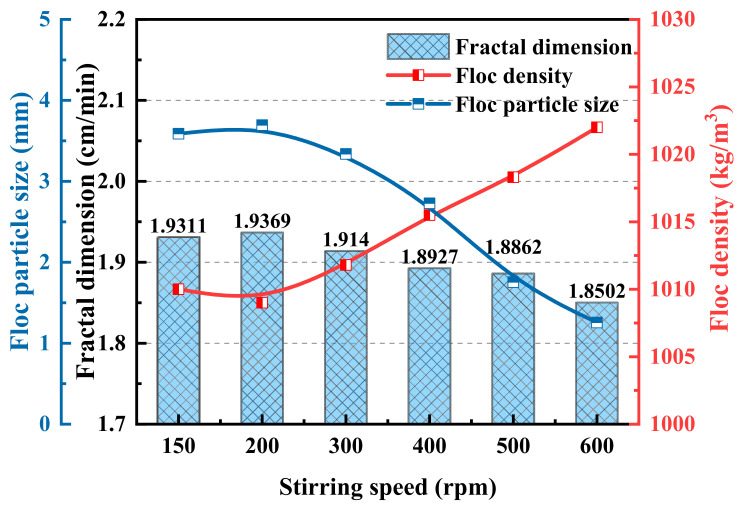
Characteristics of coal slurry flocs at different stirring speeds.

The morphology of the flocs after sedimentation at different stirring speeds was analyzed to study the strength of the chitosan coal slurry flocs. Figure 12 shows the morphology of the coal slurry flocs after sedimentation at different stirring speeds. Figure 13 shows the changes in the coal slurry floc characteristics at different stirring speeds after the image processing analysis. With the increase in stirring speed, the coal slurry floc size first increased appropriately and then gradually decreased after 300 rpm, the floc density increased, and the fractal dimension decreased, which indicates that increasing the shear strength within a certain range is beneficial for increased aggregation efficiency and floc size; however, too-high shear strength will produce a high turbulent dissipation rate, which will increase the floc crushing efficiency and decrease the floc size. When comparing the floc characteristics, the floc size was 3.694 mm at 200 rpm and 1.852 mm at 600 rpm, which decreased to approximately 1/4 of that at 200 rpm. The fractal dimension of the flocs decreased from 1.93 to 1.85, and the floc density increased from 1009 to 1022 kg/m^3^.

Primarily, floc structure and strength are issues throughout the dynamic process. For a given shear rate, the floc strength is an indication of the floc structure [47]. It has been difficult to develop a satisfactory technique to quantify floc strength. Breakage is highly dependent on the shear strength and floc strength. Floc fragmentation occasionally occurs when the generated flocs are subjected to greater shear, which may be caused by two generally accepted mechanisms of floc fragmentation: mass fragmentation and surface erosion [48]. Mass fragmentation refers to the stress on the floc surface being greater than the bond strength within the floc and the floc breaking into roughly equal-sized pieces. Surface erosion refers to the removal of small particles from the floc surface, which increases the extent of small particles. Furthermore, floc strength depends on the strength of the agent connecting the particles [5].

The chitosan dosage was 1.2 g/kg, which was too high. Under shear speeds greater than 300 rpm, the small flocs collide and adhere successfully, and the flocs become dense large flocs. Large flocs may further coalesce and become larger under the action of a strong shearing force or break down and become unable to form larger flocs. The flocs are broken in two ways: First, the generated small flocs collide with each other, fail to coalesce, and break up, or second, the edge branches of the flocs are broken, both of which make the flocs more compact. Under a certain shear force, floc agglomeration maintains a balance with fragmentation, resulting in different floc sizes under different shear strengths. After the stirring was stopped, the chitosan on the small flocs had more sites for particle adsorption, which further absorbed the microfine particles, and secondary flocculation occurred between flocs, decreasing the number of microfine particles measured by FBRM. This process cannot effectively test the specific fragmentation of chitosan agents. However, by observing the floc characteristics after sedimentation, it can be seen that the flocs formed by the chitosan agent had a certain floc strength and could still play a normal flocculation and sedimentation role under the high shear effect, although they were severely fragmented.

### 3.4. Synergistic Mechanism of Al^3+^ and Chitosan

#### 3.4.1. Results of the QCM−D Test

The above test results confirmed that the synergistic effect of AlCl_3_ and chitosan could effectively flocculate and settle coal slime water. To study the synergistic mechanism between AlCl_3_ and chitosan, QCM−D was used to study the adsorption characteristics of AlCl_3_ and chitosan on the carbon sensor surface.

**Figure 14:**
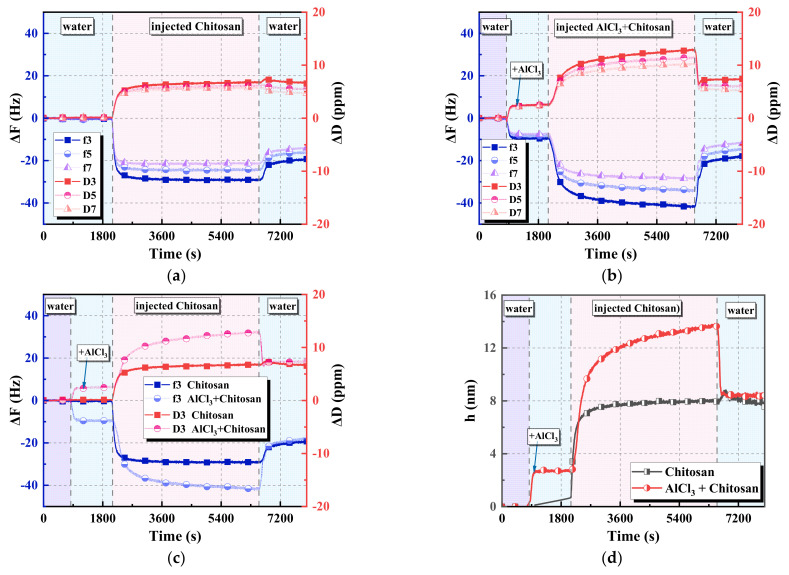
QCM−D test results for the adsorption and desorption behavior of chitosan on the carbon sensor: (**a**) trend of ΔF and ΔD for the carbon sensor fed with chitosan; (**b**) trend of ΔF and ΔD for the carbon sensor provided with AlCl_3_ followed by the AlCl_3_+chitosan mixture; (**c**) comparison of f_3_ and D_3_ under both conditions; (**d**) comparison of adsorption thickness under both conditions.

Figure 14 shows the statistical results of the adsorption and desorption dissipation of chitosan on the carbon surface in the presence and absence of AlCl_3_. As shown in Figure 14a, the adsorption of chitosan alone triggered a rapid ΔF and ΔD, with f_3_ decreasing by 28 Hz and D_3_ increasing by 6.2 ppm, indicating that chitosan rapidly adsorbed on the carbon surface. After 7 min, ΔF and ΔD remained unchanged, but the magnitude of the change slowed, indicating that the adsorption of chitosan on the carbon surface became slower. When water was introduced to rinse and desorb the chitosan, ΔF increased, ΔD decreased slightly and then stabilized, f_3_ returned to −20 Hz, and D_3_ decreased by 0.4 ppm and was maintained at 6.6 ppm. This indicated that water washed a portion of the chitosan away, the majority was adsorbed on the carbon surface, and the adsorption process was irreversible.

As shown in Figure 14b, the AlCl_3_ solution was first introduced in order to study the adsorption behavior of AlCl_3_ on the coal slurry particles after adding the AlCl_3_ coagulant to the slurry water, and ΔF and ΔD were changed. F_3_ decreased to −9.8 Hz, and D_3_ increased to 2.3 ppm after 3 min and remained stable, indicating that the adsorption of Al^3+^ ions occurred on the carbon surface and the adsorption amount was constant. Subsequently, the chitosan solution dissolved in AlCl_3_ was passed to study the adsorption of chitosan on the carbon surface when chitosan was added to the coal slurry water after adding the AlCl_3_ coagulant. ΔF continued to decrease, and ΔD continued to increase. After 20 min, the change range became slower; however, the change range was larger than that of chitosan alone, indicating that chitosan continued to adsorb on the carbon surface in the presence of AlCl_3_, and the adsorption strength was higher than that of chitosan alone. With the passage of water, in water, ΔF increased, ΔD decreased, f_3_ returned to −19.1 Hz, and D_3_ decreased by 0.4 ppm and was maintained at 7.4 ppm.

On comparing the adsorption behavior in Figure 14c,d and the adsorption thickness obtained by data fitting, it can be observed that compared with the amount of change in the energy consumption of chitosan alone on the carbon surface, the adsorption effect of the AlCl_3_ and chitosan solution on the carbon surface was stronger, the change range was larger, and continued adsorption was obvious. After 2 h of adsorption, the adsorption thicknesses of chitosan alone, the AlCl_3_ ions alone, and the AlCl_3_ and chitosan solution on the carbon sensor were 7.79, 2.77, and 13.63 nm, respectively. The desorption study was performed by feeding water. Partial desorption was performed in both cases; however, most of the agents were still adsorbed on the carbon surface, and the adsorption capacities of the two cases were similar. The QCM−D test results of the reversible adsorption of AlCl_3_ solution with a concentration of 100 mM on the carbon sensor indicated that the AlCl_3_ and chitosan solution was desorbed on the carbon surface as AlCl_3_ with some chitosan, and the desorption residues were dominated by chitosan.

#### 3.4.2. Potential Results

**Figure 15:**
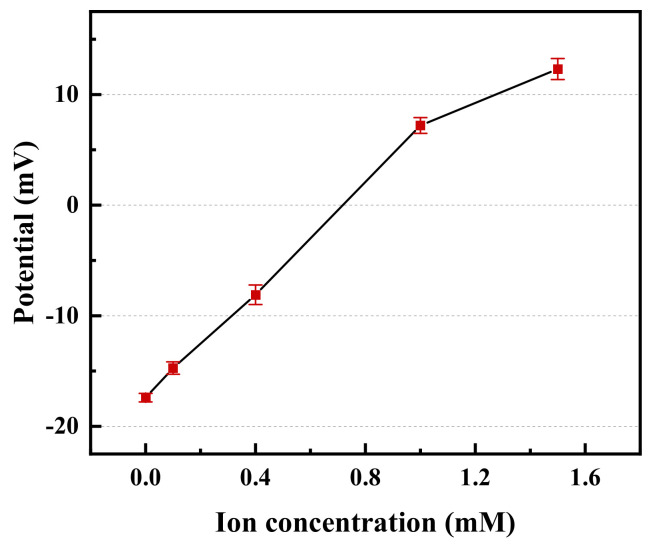
Relationship between Al^3+^ concentration and coal slurry potential.

The ζ−potential is a macroscopic reaction of the microscopic properties of the surface of the coal slurry particles that is generated by the double electric layer formed between the surface of the coal particles and the surrounding medium. Figure 15 shows the relationship between the AlCl_3_ concentration in the coal slurry water and the potential of the coal slurry particles. The surface of the coal slurry suspended particles with a strong negative charge, the ζ potential was approximately −17 mV, and there was a strong electrostatic repulsion between coal slurry particles, forming a stable, difficult-to-settle colloidal system [49]. The increase in the AlCl_3_ concentration neutralized the negative charge on the surface of the coal slurry particles [50]. The negative amount of coal slurry particles decreased, and the electrical properties changed. In this test, the concentration of Al^3+^ in coal slurry water was 0.4 mM, the ζ potential of the coal slurry particles was −7 mV, and the surface of the particles remained negatively charged. The addition of Al^3+^ neutralized the negative charge on the surface of the coal slurry, reduced the electric repulsion between particles, compressed the electric double layer, and simultaneously destroyed the hydration film on the surface of the coal slurry particles, prompting the agglomeration between micro-fine particles and reducing the difficulty for chitosan to adhere to coal slurry particles in the later stage.

Because large flocs were formed after adding chitosan to the coal slurry water, the potential measurement of coal slurry particles after the action of chitosan could not be performed well; therefore, the interaction between chitosan and coal slurry particles under different drug doses was analyzed from the perspective of the chemical properties of chitosan. In addition to its long-chain structure, chitosan also contains abundant free amino groups on its polymer chain backbone that are protonated in dilute acid solution to increase the positively charged groups of chitosan and increase the charge density of the polyelectrolyte. Chitosan behaves as a typical cationic polyelectrolyte with good flocculation performance, including effective charge neutralization and a bridging effect [51]. Furthermore, when the degree of deprotonation increases, chitosan can self-assemble from discrete states to form complex network structures [52].

**Figure 16:**
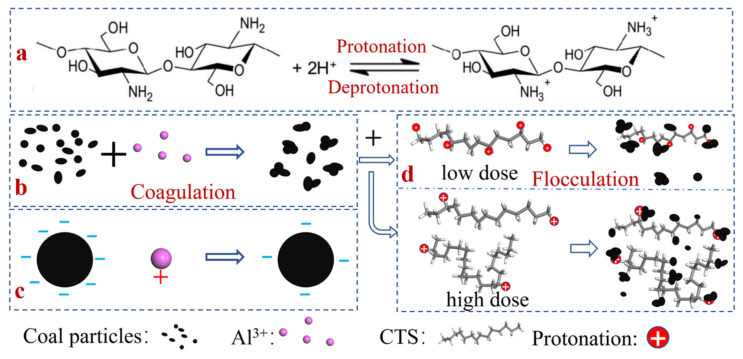
Synergistic mechanism of AlCl_3_ and chitosan during the coagulation–flocculation processes: (**a**) protonation and deprotonation of chitosan, (**b**) coagulation process by the action of AlCl_3_, (**c**) the action of Al^3+^ reduces the surface potential of coal particles, and (**d**) flocculation by the action of different chitosan doses.

Figure 16 shows a schematic diagram of the synergistic mechanism of AlCl_3_ and chitosan during coagulation–flocculation. Many microparticles remained in the supernatant when chitosan was added to the coal slurry water for flocculation and sedimentation, and the synergistic effect of AlCl_3_ was added to clarify the coal slurry water. While AlCl_3_ initially agglomerated the coal slime particles, the hydrolysis of Al^3+^ promoted the protonation of chitosan. For a low chitosan dosage, the synergistic effect of AlCl_3_–chitosan improved the electric neutralization ability and adsorption bridging ability of coagulation, which made the floc structure compact and increased the floc size. For the high chitosan dosage, the protonation of chitosan was weakened, the chitosan self-assembled into a mesh structure, and flocculation was mainly based on adsorption and bridging. The formed flocs increased in size and pore size, which is consistent with the floc morphology in the flocculation sedimentation test.

## 4. Conclusions

The addition of chitosan can significantly improve the flocculation–sedimentation and dewatering effects of coal slurry water. Chitosan can meet the pharmaceutical requirements for the solid–liquid separation of coal wastewater and can replace PAM as an environmentally friendly and efficient flocculant. At a chitosan dose of 1.0 g/kg, the settling velocity of coal wastewater reached 65 cm/min, clarity was the best, light transmittance was 58.7%, floc size reached 1.83 mm, floc density was 1014 kg/m^3^, fractal dimension was 1.8862, filtration rate of coal slime was improved, and average mass specific resistance of filtration was reduced.

With an increase in the chitosan dosage, the number of residual fine particles in the upper clarification layer first decreased and then increased, and the size of coal slime flocs increased in direct proportion to the chitosan dosage. Simultaneously, the pores and edge branches of the flocs increased, and the floc density decreased. The slime flocs formed using chitosan had a certain strength. Although an increase in shear strength results in an increase in coal slime flocculation, the final secondary flocculation is beneficial for the reduction of fine particles.

Compared with the amount of change in the energy consumption of chitosan alone on the carbon surface, the AlCl_3_ and chitosan solution showed stronger adsorption on the carbon surface, with greater variation and continuous adsorption. At a low chitosan dosage, Al^3+^ hydrolysis promoted the protonation of chitosan, and the synergistic effect of AlCl_3_––chitosan improved the electric neutralization ability and adsorption bridging ability of coagulation, which made the floc structure compact and increased the floc size. For the high chitosan dosage, the protonation of chitosan was weakened, and the chitosan self-assembled into a mesh structure, flocculation was mainly based on adsorption and bridging, and the floc and pore sizes increased.

Therefore, chitosan can replace PAM as an environmentally friendly and efficient flocculant for the solid–liquid separation of coal wastewater, and this study provides a new approach for the selection of flocculants for coal wastewater treatment.

## Figures and Tables

**Table 1:** Proximate and ultimate analyses of the samples (%) ^a^.

Proximate Analysis/wt%				Ultimate Analysis/wt%				
V_ad_	A_ad_	M_ad_	FC_ad_	C	H	N	S	O
45.9	11.21	1.76	41.13	59.72	5.67	1.03	0.43	33.15

^a^ M_ad_, moisture content; V_ad_, volatile content; FC_ad_, fixed carbon content; and A_ad_, ash content.

## Data Availability

Not applicable.
